# Revealing Unknown Protein Structures Using Computational Conformational Sampling Guided by Experimental Hydrogen-Exchange Data

**DOI:** 10.3390/ijms19113406

**Published:** 2018-10-31

**Authors:** Didier Devaurs, Dinler A. Antunes, Lydia E. Kavraki

**Affiliations:** Department of Computer Science, Rice University, 6100 Main St, Houston, TX 77005, USA; devaurs@rice.edu (D.D.); dinler@rice.edu (D.A.A.)

**Keywords:** protein structure, protein conformational sampling, hydrogen exchange, mass spectrometry, nuclear magnetic resonance

## Abstract

Both experimental and computational methods are available to gather information about a protein’s conformational space and interpret changes in protein structure. However, experimentally observing and computationally modeling large proteins remain critical challenges for structural biology. Our work aims at addressing these challenges by combining computational and experimental techniques relying on each other to overcome their respective limitations. Indeed, despite its advantages, an experimental technique such as hydrogen-exchange monitoring cannot produce structural models because of its low resolution. Additionally, the computational methods that can generate such models suffer from the curse of dimensionality when applied to large proteins. Adopting a common solution to this issue, we have recently proposed a framework in which our computational method for protein conformational sampling is biased by experimental hydrogen-exchange data. In this paper, we present our latest application of this computational framework: generating an atomic-resolution structural model for an unknown protein state. For that, starting from an available protein structure, we explore the conformational space of this protein, using hydrogen-exchange data on this unknown state as a guide. We have successfully used our computational framework to generate models for three proteins of increasing size, the biggest one undergoing large-scale conformational changes.

## 1. Introduction

A protein’s function is known to be modulated by changes in the protein’s three-dimensional structure [1]. Understanding how a protein shifts between conformations is essential to treat or prevent diseases caused by its dysfunction [2]. This requires gathering information about the protein’s conformational space, i.e., the space of all possible states in which the protein can be [3]. Some information can be obtained experimentally, using techniques such as X-ray crystallography, which has produced many of the structures reported in the Protein Data Bank (PDB) [4]. To obtain additional information, experimental techniques have been complemented by various computational methods, such as molecular dynamics [5]. However, when dealing with large proteins or molecular complexes, both experimental techniques and computational methods suffer from their respective limitations. Therefore, studying large proteins and molecular complexes remains a critical challenge for structural biology.

At one end of the experimental spectrum, techniques such as X-ray crystallography achieve very high accuracy, yielding atomic-resolution structural models, but they present strong limitations in terms of cost and applicability. At the other end of the spectrum, techniques such as hydrogen-exchange monitoring are increasingly less expensive and easier to implement [6]. Hydrogen-exchange monitoring can be performed using mass spectrometry or nuclear magnetic resonance spectroscopy. This experimental technique provides valuable indirect information about a protein’s structure and dynamics, despite its low resolution [7]. Its advantages have led to numerous applications for the analysis of protein structure and conformational changes, as well as molecular interactions [8]. However, as this experimental technique cannot produce structural models on its own, computational methods are needed to complement it and generate these models.

Monte Carlo-based conformational sampling [9] and molecular dynamics [10,11,12] have been used to complement hydrogen-exchange monitoring. Going in a different direction, our work leverages robotics-inspired algorithms to model and analyze protein structure, by implementing efficient conformational sampling methods that allow exploring a protein’s conformational space [13]. In this context, we use a computational tool, named Structured Intuitive Move Selector (SIMS), which integrates sampling-based path planning algorithms [14,15] with the well-established Rosetta library for protein modeling [16,17]. However, similar to other computational methods for conformational sampling, SIMS suffers from the curse of dimensionality when applied to large proteins. To address this issue, a common strategy involves using experimentally-obtained data to guide the conformational sampling [18,19,20]. Following this common idea, we have recently proposed a computational framework in which SIMS can be guided by experimental hydrogen-exchange data [21].

In previous work, we had only applied our computational framework to the conformational exploration of the native state of proteins [21,22]. The main objective of this application was to analyze and refine the experimental hydrogen-exchange data itself and to study the conformational variability of a protein’s native state. In this paper, we apply our framework to a more challenging problem, that of generating an atomic-resolution structural model for an unknown protein state. The typical scenario to which this work applies is the following: Assuming that an atomic-resolution model of a given protein state exists, how do we obtain a structural description of another protein state for which only hydrogen-exchange data are available? Answering this question requires exploring large regions of this protein’s conformational space, beyond a specific state.

Here, we present the results we have obtained when applying our computational framework, based on the idea of guiding SIMS’ conformational search with experimental hydrogen-exchange data [21], to three proteins of increasing size. First, we validate our methodology by reproducing a known conformation of a small protein, interleukin-8. Then, we generate a structural model for the unbound state of a medium-sized protein, the vitamin D receptor, for which structures have been reported in the PDB only for its bound state. Finally, we create an atomic-resolution structural model for the native state of a large protein, the complement protein iC3b, which has only been described by medium-resolution structures.

## 2. Results

We have used our computational framework involving our efficient conformational sampling tool, SIMS (see Section 4.4) [21], to explore the conformational space of three proteins of different sizes. By guiding the conformational exploration with experimental hydrogen-exchange data collected for these proteins, we have generated atomic-resolution structural models describing the states in which these proteins were when these data were collected.

### 2.1. Interleukin-8

**Figure 1:**
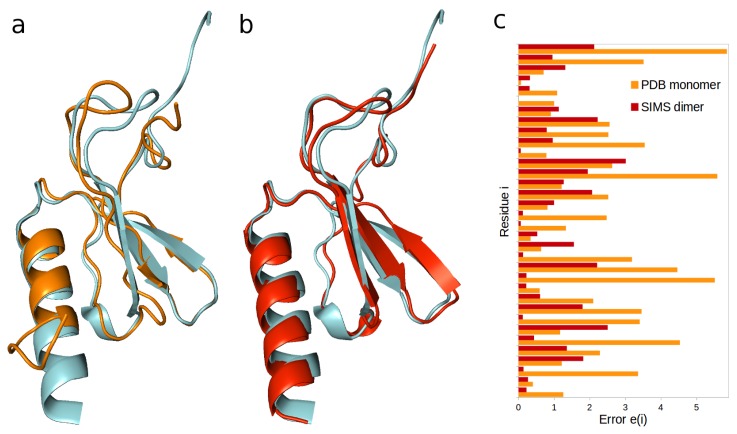
Reproducing the interleukin-8 (IL-8) dimer. (**a**) The conformation of the non-natural IL-8 monomer (colored orange) differs from the conformation of the monomer within the wild-type IL-8 homodimer (colored blue). (**b**) The conformation of IL-8 produced by SIMS (colored red) is very similar to that of the wild-type IL-8 (colored blue). (**c**) Differences between structurally-derived and experimental hydrogen-exchange data (unitless error *e(i)*, for each residue *i*) are significantly lower when these data are derived from the conformation produced by SIMS (colored red) than when they are derived from the non-natural monomer’s conformation (colored orange).

First, we used interleukin-8 (IL-8) as a model system to validate our approach. Since a hydrogen-exchange monitoring experiment had been performed on the wild-type dimer form (see Section 4.5.1) [23], we decided to verify whether we could produce this dimer’s conformation (PDB ID 1IL8) by running a conformational search with SIMS, guided by these hydrogen-exchange data. As a starting point for this conformational search, we used an artificial model for the dimer form, built using two copies of the non-natural monomer form (PDB ID 1IKL). The conformation of this monomer form of IL-8 differs from the conformation of the dimer form, mostly in that its C-terminus is partly disordered (see Figure 1a): the all-atom root-mean-squared deviation (RMSD) between these two conformations is 4.6 Å. To perform the conformational search, we ran SIMS five times. Except for the first one, each run of SIMS was biased by the differences between the structurally-derived and the experimental hydrogen-exchange data (see Section 4.4).

As a result of the search, we obtained a conformation for the dimer that fits the experimental hydrogen-exchange data well (see Figure 1c). The average difference between the structurally-derived and the experimental hydrogen-exchange data equals 0.99, when deriving these data from the conformation produced by SIMS. In comparison, when deriving these data from the artificial model used as a starting point for the conformational search, the average difference equals 2.27. In addition, the conformation produced by SIMS (see Supplementary File S1) is very similar to the conformation of the wild-type IL-8 (see Figure 1b): the all-atom RMSD between these two conformations is 2.1 Å.

### 2.2. Vitamin D Receptor

We then applied our computational framework to the analysis of the ligand-binding domain of the vitamin D receptor (VDR-LBD). Although numerous structural models of VDR-LBD bound to various ligands have been reported in the PDB, no model is available for its unbound form (i.e., without ligand). However, hydrogen-exchange monitoring experiments have been performed on the unbound VDR-LBD by the Griffin group at the Scripps Research Institute (see Section 4.5.2) [24]. Therefore, we decided to generate a structural model for this unbound form of VDR-LBD, by using SIMS to perform a conformational exploration guided by these experimental data. The starting point of the exploration was an artificial structure of the unbound VDR-LBD built by removing the ligand from an available VDR-LBD complex (PDB ID 3P8X). We ran SIMS five times: every run (except the first one) was biased by the differences between the structurally-derived and the experimentally-obtained hydrogen-exchange data.

**Figure 2:**
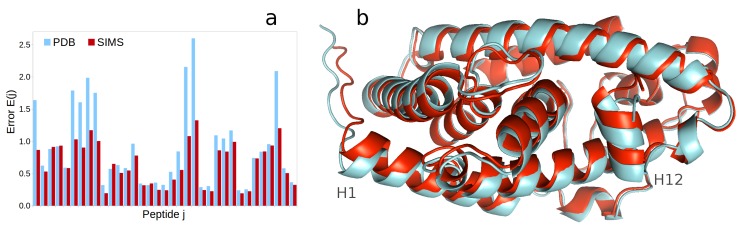
Revealing the conformation of the unbound form of the vitamin D receptor’s ligand-binding domain (VDR-LBD). (**a**) Differences between structurally-derived and experimental hydrogen-exchange data (unitless error *E(j)*, for each peptide *j*) are significantly lower when these data were derived from the conformation produced by SIMS for the unbound VDR-LBD (colored red) than when they were derived from the conformation of the bound VDR-LBD reported in the PDB (colored blue). (**b**) The conformation of the unbound VDR-LBD produced by SIMS (colored red) differs from that of the bound VDR-LBD from the PDB (ID 3P8X, colored blue) mostly in the positions of α-helices H1 and H12.

The exploration produced a conformation of the unbound form of VDR-LBD (see File S1) that fits the experimental hydrogen-exchange data well (see Figure 2a). The average difference between the structurally-derived and experimental hydrogen-exchange data is 0.66. On the other hand, when deriving hydrogen-exchange data from the artificial model used to seed the conformational exploration, the average difference is 0.92. With respect to the backbone structure, the conformation produced by SIMS is similar to the conformation of VDR-LBD bound to an agonist ligand, such as in the complex under PDB ID 3P8X. However, we observe that removing the ligand yields a slightly more compact conformation: the radius of gyration of the SIMS conformation is 18.47, as compared to 18.68 for the bound form of VDR-LBD under PDB ID 3P8X. The largest conformational changes are observed for helices H1 and H12, which both come closer to the core of VDR-LBD (see Figure 2b). These conformational changes are greater than what could be attributed to the natural variability observed between various crystal structures of the bound form of VDR-LBD. Indeed, the Cα RMSD between the SIMS conformation and the 3P8X conformation is 0.64 Å, which is almost double the largest Cα RMSD observed between all available crystal structures of the bound VDR-LBD.

### 2.3. Complement Protein iC3b

Finally, we used our computational framework to generate an atomic-resolution structural model for the complement protein iC3b. Due to its conformational variability in solution, no high-resolution structural model of iC3b has ever been reported. However, two competing medium-resolution models have been obtained by different research groups, using electron microscopy (EM): one model suggesting an extended conformation and the other suggesting a compact one (see Section 4.5.3). Additionally, hydrogen-exchange monitoring experiments have been performed on iC3b in its native state by the Lambris group at the University of Pennsylvania [25]. Their study concludes that the EM model featuring an extended conformation of iC3b is more consistent with their data than the other model. We decided to computationally confirm their conclusion, by performing an exploration of iC3b’s conformational space using SIMS guided by their hydrogen-exchange data. As a starting point for this conformational exploration, we built a hypothetical atomic-resolution model of iC3b by removing from the crystal structure of its parent protein, C3b (PDB ID 2I07), the residues corresponding to the fragment C3f (see Section 4.5.3). Starting from this model, we ran SIMS 20 times: every run but the first one was biased by the differences between the structurally-derived and the experimental hydrogen-exchange data.

**Figure 3:**
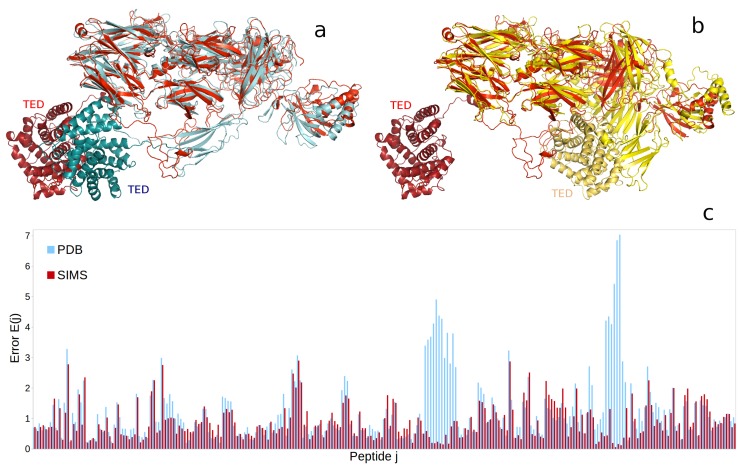
Uncovering the conformation of the complement protein iC3b. (**a**) Comparison between the conformation of iC3b produced by SIMS (colored red) and the hypothetical model built using the crystal structure of C3b reported in the PDB (ID 2I07, colored blue). In both conformations, the thioester-containing domain (TED), at the bottom left (colored in darker shades), is far from the protein’s core, (**b**) The conformation of C3 (PDB ID 2A73, colored yellow) is more compact, and the TED (colored dark yellow) is close to the protein’s core. (**c**) Differences between structurally-derived and experimental hydrogen-exchange data (unitless error E(j), for each peptide *j*) are significantly lower when these data are derived from the conformation of iC3b produced by SIMS (colored red) than when they are derived from iC3b’s hypothetical model (colored blue).

As a result of the conformational exploration performed by SIMS, we obtained a conformation of iC3b (see Supplementary File S1) that fit the experimental hydrogen-exchange data well (see Figure 3c). The average difference, across all peptides, between the structurally-derived and the experimental hydrogen-exchange data is 0.89 when deriving these data from the conformation produced by SIMS and 1.26 when deriving them from the hypothetical model used as the starting point of the conformational exploration. In addition, we observe that the conformation of iC3b produced by SIMS is an extended one (see Figure 3a) and is therefore consistent with the extended EM model of iC3b [26]. This conformation is significantly different from that of the complement protein C3 (see Figure 3b), which is supposed to be similar to the compact EM model of iC3b [27]. Therefore, our computational study corroborates the results of the experimental study in [25] and extends it by producing an atomic-resolution structural model of iC3b’s native state.

## 3. Discussion

We have recently proposed a framework in which our computational method for protein conformational sampling, called Structured Intuitive Move Selector (SIMS), can be guided by experimental hydrogen-exchange data (see Section 4.4) [21]. In previous work, we had only used this framework to explore the native state of proteins for the purpose of analyzing and refining the experimental data. In this paper, we have presented a more challenging application of our computational framework: generating an atomic-resolution structural model for an unknown protein state. We have reported results we obtained when applying our framework to three proteins of increasing size. Before making any prediction, we validated our approach using known conformations of a small protein: interleukin-8 (IL-8). Starting from the conformation of a non-natural monomer of IL-8, we ran a conformational exploration guided by the hydrogen-exchange data of the IL-8 dimer, and we successfully reproduced this dimer’s conformation.

Then, we used our computational framework to generate a high-resolution structural model for the unbound state of the ligand-binding domain of the vitamin D receptor (VDR-LBD). Starting with the conformation of VDR-LBD bound to an agonist ligand obtained from the PDB, we ran a conformational search guided by hydrogen-exchange data collected for the unbound VDR-LBD. Eventually, we obtained a conformation of VDR-LBD that is quite similar to its bound conformation, except for the position of helices H1 and H12, which moved closer to the molecule’s center. Therefore, our results show that conformational changes experienced by VDR-LBD during binding cannot be likened to the “mousetrap mechanism” that was hypothesized for other proteins of the nuclear receptor family [28]. Indeed, it is believed that a better model to explain VDR-LBD’s conformational variability involves shifts in its conformational ensemble, triggered by the binding of an agonist (favoring co-activation) versus an antagonist (favoring co-repression) [29].

Finally, we created an atomic-resolution structural model for the complement protein iC3b with our computational framework. This model was produced by an exploration of iC3b’s conformational space guided by the hydrogen-exchange data of its native state and starting from a conformation built using the crystal structure of its parent protein, C3b. As a result of this exploration, the conformation we obtained for iC3b was consistent with one of two competing medium-resolution models obtained through electron microscopy [26,27]. The conclusion of our computational analysis about the likelihood of these two models is similar to that reached by a previous experimental study of iC3b’s native state [25]. Our work extends that study by providing the first plausible atomic-resolution model for the complement protein iC3b.

In conclusion, the results we report in this paper establish evidence that a computational conformational sampling method guided by experimental hydrogen-exchange data can generate a structural model for a protein state described only by this low-resolution data. Note that the conformational exploration requires an atomic-resolution structural model of the protein of interest as a starting point; this can be the structure of another state of that protein reported in the PDB or a model built by modifying the structure of an analogous protein. We have validated our methodology with a small protein and successfully applied it to reveal unknown structures of a medium-sized protein and a large protein subject to large-scale conformational changes.

As future work, we plan to expand the application field of our computational framework, by generating structural models for unknown non-native states, such as folding intermediates, of a protein. Contrary to the protein states we have studied in this paper, protein non-native states are not necessarily well characterized by existing force fields and scoring functions, which have been developed to favor native arrangements and folds. Therefore, our main challenge will be to ensure that our methodology can produce conformations of non-native states described only by experimental hydrogen-exchange data without being hampered by biases in the scoring function. The overall objective of our work will remain to help researchers studying protein structure by creating atomic-resolution models that cannot be obtained experimentally.

## 4. Materials and Methods

### 4.1. Hydrogen Exchange in Proteins

Hydrogen exchange (HX) is the chemical phenomenon by which hydrogen atoms in a protein are naturally replaced by hydrogens (or their isotopes) in the surrounding solvent [30]. If a protein is placed in a heavy water solution (D${}_{2}$O), the hydrogen in the protein will exchange with the deuterium in the solvent. Using experimental techniques that can capture differences between hydrogen isotopes, one can thus monitor hydrogen exchange [31]. Nuclear magnetic resonance (NMR) spectroscopy was the original technique used to measure hydrogen exchange, in so-called HX-NMR experiments [32]. Later on, thanks to its significant advantages, mass spectrometry (MS) became more popular and led to the conducting of numerous HX-MS experiments [33].

Experiments monitoring hydrogen-exchange can only record the exchange of backbone amide hydrogens and can therefore produce only one value per amino acid, except for proline residues and for the N-terminus of the polypeptide chain, which cannot be monitored. The hydrogen-exchange rate of a given amino acid is known to vary up to several orders of magnitude, depending on pH and temperature [34]. The intrinsic exchange rate of a residue in an unstructured peptide, $k^{\mathrm{int}}$, is only affected by its adjacent amino acids and can be predicted [35,36]. The experimentally-observed exchange rate of a residue in a protein, $k^{\mathrm{obs}}$, is additionally influenced by its solvent accessibility and the protein’s structure [37]. Indeed, residues that are involved in α-helices or β-sheets, as well as residues located in the protein’s core are more protected from hydrogen exchange than other residues. To quantify the extent to which residues are protected from hydrogen exchange in a protein, one can define the protection factor of every amino acid *i* by $P_{i}=k_{i}^{\mathrm{int}}\;/\;k_{i}^{\mathrm{obs}}$.

In HX-NMR experiments, results are reported at the residue level, usually as a list of protection factors, but obtaining a good coverage of the protein is challenging [31]. On the other hand, in HX-MS experiments, measurements are acquired at the peptide level, but often yield a good coverage of the protein. HX-MS results are usually reported as a set of deuterium-uptake kinetic curves for various peptides, typically 6–20 amino acids in length [32].

### 4.2. Hydrogen Exchange Derived from Protein Structure

The levels of hydrogen exchange undergone by different parts of a protein are known to be partly influenced by its local structure. Several theoretical models have been proposed to define a relationship between a protein’s conformation and the corresponding hydrogen exchange [21]. Among these models, we have chosen to use the one that performed best in a recent comparative study [38]. This model relies on a phenomenological expression approximating the protection factors of the protein’s residues [9]. It has been used to computationally derive hydrogen exchange from protein structure in several studies [10,11,12,21,22,39].

This model is based on the assumption that protection from hydrogen exchange arises from the involvement of residues in hydrogen bonds and from the atom packing around residues. The protection factor of residue *i* in conformation *C*, $P_{i}\left(C\right)$, can be derived from the phenomenological expression:

(1) $$\ln P_{i}\left(C\right)=\beta^{h}\;N_{i}^{h}\left(C\right)+\beta^{c}\;N_{i}^{c}\left(C\right)$$

where $N_{i}^{h}\left(C\right)$ is the number of hydrogen bonds involving residue *i* and $N_{i}^{c}\left(C\right)$ is the number of atom contacts (quantifying atom packing) that involve residue *i*. Parameters $\beta^{h}$ and $\beta^{c}$ were estimated by fitting experimental hydrogen-exchange data from several proteins: $\beta^{h}=2$ and $\beta^{c}=0.35$ [10]. The number of hydrogen bonds, $N_{i}^{h}\left(C\right)$, is the number of backbone oxygens in any residue (except residues i−2, …, i+2) within a cutoff distance of 2.4 Å from the amide hydrogen of residue *i*. The number of atom contacts, $N_{i}^{c}\left(C\right)$, is the number of heavy atoms in any residue (except residues i−2, …, i+2) within a cutoff distance of 6.5 Å from the amide hydrogen of residue *i*.

The residues’ protection factors derived from (1) can be directly compared to protection factors obtained in an HX-NMR experiment. On the other hand, in the case of an HX-MS experiment, one has to generate deuterium-uptake curves of peptides that can be compared to the experimental data. For that, we assume that a residue’s deuterium uptake follows pseudo-first-order kinetics [32,34,40]. Since $P_{i}=k_{i}^{\mathrm{int}}\;/\;k_{i}^{\mathrm{obs}}$, the fraction of deuterium incorporated by residue *i* at time *t* is thus:

(2) $$d_{i}\left(t\right)=1-\exp\left(-k_{i}^{\mathrm{obs}}\;t\right)=1-\exp\left(-\left(k_{i}^{\mathrm{int}}/P_{i}\right)\;t\right)\;.$$

Then, the deuterium uptake of peptide *j* is calculated as an average over the residues it contains [12]. Note that we systematically exclude from the average the first two amino acids of the peptide because of an important phenomenon known as back-exchange [32,40]. More details on how to derive HX-MS data from a protein conformation are presented in [21].

### 4.3. Goodness-of-Fit between Structurally-Derived and Experimental Hydrogen-Exchange Data

Using the hydrogen-exchange prediction model, one can derive hydrogen-exchange data from a protein’s conformation and compare it to experimental data. To assess the goodness-of-fit between structurally-derived and experimental hydrogen-exchange data, one can use the following method:

- When dealing with HX-NMR data, one can obtain a histogram of errors by computing, for every residue *i*, the unitless error $e\left(i\right)=|\ln P_{i}^{\mathrm{der}}-\ln P_{i}^{\mathrm{obs}}|$, where $P_{i}^{\mathrm{der}}$ is the structurally-derived protection factor and $P_{i}^{\mathrm{obs}}$ is the experimentally-observed protection factor. One can also aggregate this histogram into an average error over all relevant residues.
- In the case of HX-MS data, one can obtain a histogram of errors by computing, for each peptide *j*, the unitless error $E\left(j\right)=\sum_{t\in T}\left|D_{j}^{\mathrm{der}}\left(t\right)-D_{j}^{\mathrm{obs}}\left(t\right)\right|$, where *T* is the list of experimental time points in the HX-MS experiment, $D_{j}^{\mathrm{der}}\left(t\right)$ is the structurally-derived deuterium uptake at time *t* and $D_{j}^{\mathrm{obs}}\left(t\right)$ is the experimentally-observed deuterium uptake at time *t*. One can also aggregate this histogram into an average error over all peptides.

### 4.4. Efficient Conformational Sampling of Protein Structure

The computational framework we use to explore a protein’s conformational space is called Structured Intuitive Move Selector (SIMS) [13]. This framework integrates robotics-inspired sampling algorithms with the well-known Rosetta library for protein modeling [16,17]. In SIMS, a protein model involves only backbone dihedral angles, and this model is manipulated in a multi-resolution fashion during conformational sampling; side chains are not modeled explicitly, but optimized by Rosetta instead, every time a new conformation is created. The exploration starts from a known protein conformation, such as a crystal structure reported in the PDB, and aims at producing new low-energy conformations by perturbing existing ones. The so-called protein moves that allow creating new conformations are: loop sampling, rigid-body motion, dihedral angle sampling and short energy minimization. Our methodology is similar to Monte Carlo techniques and differs from molecular dynamics methods, in that it does not simulate a protein’s behavior over time, but only explores its conformational space. Using robotics-inspired techniques allows performing this exploration efficiently, by incrementally building a graph structure whose nodes are conformations and whose edges represent possible transitions between them [13].

The methodology we use to generate conformations producing a good fit to the experimental hydrogen-exchange data is the following: First, we use SIMS to perform an unbiased conformational exploration starting from a known structure of a protein. From the ensemble of conformations generated by SIMS, we determine which one provides the best fit to the experimental hydrogen-exchange data, using the aforementioned methods and equations. If we estimate that a good fit has been reached (see Section 4.3) and if no further improvement is observed, no additional run of SIMS is performed. If we are not satisfied with the goodness-of-fit, we run SIMS again, using the largest differences between structurally-derived and experimental hydrogen-exchange data as a sampling bias. More precisely, protein regions where these differences are the largest are assigned higher probabilities to be sampled. Note that a single run of SIMS lasts 24 h on four threads of a 3.6-GHz Intel i7-4790 quad-core CPU. We repeat this iterative process of running SIMS and analyzing the generated conformations until no significant improvement in the goodness-of-fit is observed. For more details on this methodology, the interested reader is referred to [21].

### 4.5. Studied Proteins and Experimental Hydrogen-Exchange Data

#### 4.5.1. Interleukin-8

Interleukin-8 (IL-8) is a small protein composed of 72 amino acids. It is a chemokine that is known to be involved in acute and chronic inflammatory diseases [41]. Its structure consists of a disordered N-terminus, followed by a triple-stranded β-sheet and a C-terminal α-helix. Since IL-8 naturally dimerizes at a high concentration in solution, mostly structural models of the homodimer have been reported in the protein data bank (PDB ID 1IL8 and 3IL8) [42,43]. Obtaining a structural model of the monomer was only made possible by the discovery that a specific chemical alteration could prevent dimer formation [41]. The structure of this chemically-synthesized monomer (PDB ID 1IKL) is similar to that of the wild-type monomer within the homodimer, but differences exist. The main difference is that the C-terminal residues 67–72 are disordered in the monomer form, whereas they are part of the α-helix in the dimer form.

A HX-NMR experiment performed on the wild-type homodimer form of IL-8 produced free energies of stabilization ($\Delta G_{HX}$) for 35 of its amino acid residues [23]. Protection factors $P_{i}$ were derived from free energies $\Delta G_{i}$ using the following equation:

(3) $$\Delta G_{i}=R\;T\;\ln P_{i}$$

where R=0.0019872036 kcal K${}^{-1}$ mol${}^{-1}$ is the gas constant and T=298.15 K is the experimental temperature.

#### 4.5.2. Vitamin D Receptor

The human vitamin D receptor (VDR) is a medium-sized protein composed of 427 amino acids, from the nuclear receptor superfamily. It can be functionally divided into four regions: a short amino terminus, a DNA-binding domain, a hinge domain and a ligand-binding domain (between residues 124 and 427) [44]. No structure of the whole protein has been so far reported in the PDB. However, numerous studies have led to crystal structures of VDR’s ligand-binding domain (LBD), after the discovery that a mutant VDR lacking an undefined hinge loop (between residues 165 and 215) could be crystallized [45]. These studies also found that, as this loop is a poorly-conserved insertion domain, deleting it would not impact VDR’s overall structure or function. The molecule resulting from this deletion, which we simply refer to as VDR-LBD in this paper, is composed of 253 amino acids. Its structure consists of 12 α-helices (usually denoted by H1–H12) folded into a three-layered antiparallel sandwich forming a hydrophobic binding pocket [44].

The conformation of VDR is known to be stabilized by ligand binding [45]. As a result, only crystal structures of VDR-LBD in complex with various ligands have been reported in the PDB. More specifically, these ligands are all agonists: they are known to render VDR receptive to co-activator binding. To the best of our knowledge, there is no reported structure of VDR-LBD in complex with an antagonist, i.e., a ligand that would render VDR receptive to co-repressor binding. Among the 38 crystal structures we obtained from the PDB, we chose the one with the highest Verify-3D score: 3P8X [46]. The conformations of VDR-LBD in the other crystal structures are very similar to this one: the largest Cα root-mean-squared deviation (RMSD) we observed was only 0.35 Å. To construct a hypothetical model of the unbound form of VDR-LBD (i.e., the form without ligand), we deleted the ligand from the crystal structure with PDB ID 3P8X.

Numerous HX-MS experiments have been performed on VDR-LBD [47,48] by Dr. Patrick Griffin’s team at the Scripps Research Institute, including experiments on its unbound form [24]. They accepted to share with us the HX-MS data they had collected during one such experiment [29]. They consist of deuterium-uptake curves for 35 peptides extracted from the unbound form of VDR-LBD in its native state, alone in solution. The sequence coverage achieved by combining all these peptides is about 89%. The HX-MS data were averaged over three replicate experiments lasting 1 h each, with five intermediate time steps at: 10 s, 30 s, 1 min, 5 min and 15 min.

#### 4.5.3. Complement Protein iC3b

As part of the immune system, the complement system is known to modulate various immune responses against pathogens, but also to sometimes drive immune and inflammatory diseases [49]. At the center of the complement system, the complement component C3 is the point of convergence for all complement activation pathways and the driving force behind complement response amplification. C3 is a large protein composed of more than 1600 amino acids. It consists of two chains forming 13 domains, among which eight homologous domains constitute the macroglobulin (MG) core of the molecule [50]. C3 is known to undergo large conformational changes upon activation by the C3 convertases, but the MG core largely remains unchanged. The domains that experience the largest displacements are the thioester-containing domain (TED) and the so-called CUB domain, which connects the TED to the MG core. When C3 is activated into C3b as the result of the proteolytic cleavage of the small fragment C3a, the TED and CUB domain extend away from the MG core to allow for C3b to attach to a cell surface. Several structural models of C3 and its various fragments, such as C3b, have been reported in the PDB [50].

The cleavage of the small fragment C3f in the CUB domain of C3b results in the formation of iC3b, another large complement protein composed of more than 1500 amino acids. This cleavage also leads to an increase in the flexibility of the TED and CUB domain, which has hampered all efforts to obtain a high-resolution structural model for iC3b [50]. Only two medium-resolution structural models that appear mutually inconsistent have been produced by 3D electron microscopy (EM) [26,27]. The first model implies that the TED and CUB domain extend even further away from the MG core [26], while the second model suggests a more compact structure, similar to that of C3 [27]. To construct a hypothetical high-resolution model of iC3b, we used a crystal structure of C3b in its native state (PDB ID 2I07) [51], from which we removed the residues corresponding to C3f. We then used this model as the starting point of a conformational exploration performed by SIMS.

The team of Dr. John Lambris at the University of Pennsylvania has performed numerous HX-MS experiments on the complement protein C3 and its fragments [52,53,54], the most recent one targeting iC3b [25]. The results of their experiments on iC3b’s native state suggest that the extended EM structural model [26] is the most realistic one. They kindly accepted to give us access to their HX-MS data on iC3b’s native state [55]. Their data consist of deuterium-uptake curves for 264 peptides, leading to a sequence coverage of 91%. These data were averaged over two replicate experiments with eight time points: 10 s, 30 s, 100 s, 5 min, 17 min, 50 min, 167 min and 7 h [25]. We used these data to bias the aforementioned exploration of iC3b’s conformational space, to try and confirm which one, among the two competing EM models, best describes i3Cb’s native state.

## Supplementary Materials

The following is available online at http://www.mdpi.com/1422-0067/19/11/3406/s1: File S1: Archive containing the structural models (in PDB format) generated by SIMS for all studied proteins.

## Abbreviations

The following abbreviations are used in this manuscript:

EM	electron microscopy	NMR	nuclear magnetic resonance
HX	hydrogen exchange	PDB	Protein Data Bank
IL-8	interleukin-8	RMSD	root-mean-squared deviation
LBD	ligand-binding domain	SIMS	Structured Intuitive Move Selector
MG	macroglobulin	TED	thioester-containing domain
MS	mass spectrometry	VDR	vitamin D receptor
